# Exploring the impact of chronic urticaria profile as a key predictor of alexithymia: A cross‐sectional study

**DOI:** 10.1002/clt2.70075

**Published:** 2025-07-04

**Authors:** Ivan Cherrez Ojeda, Simon Francis Thomsen, Ana M. Gimenez‐Arnau, Jennifer Astrup Sørensen, Hermenio Lima, Kiran Godse, Carole Guillet, Luis Escalante, Astrid Maldonado, Gonzalo Federico Chorzepa, Blanca Morfin‐Maciel, Jose Ignacio Larco Sousa, Erika de Arruda‐Chaves, Abhishek De, Daria Fomina, Anant Patil, Roberta Jardim Criado, Luis Felipe Ensina, Solange O. R. Valle, Rosana Câmara Agondi, Herberto Chong Neto, Nelson Rosario, German Dario Ramon, Marco Faytong‐Haro, Isabel Ogueta, Ivan Tinoco Moran, Jennifer Donnelly, Emek Kocatürk, Anna Zalewska‐Janowska, Karla Robles‐Velasco

**Affiliations:** ^1^ Institute of Allergology Charité—Universitätsmedizin Berlin Corporate Member of Freie Universität Berlin and Humboldt‐Universität zu Berlin Berlin Germany; ^2^ Universidad Espiritu Santo Samborondon Ecuador; ^3^ Respiralab Research Group Guayaquil Ecuador; ^4^ Department of Dermatology Bispebjerg Hospital University of Copenhagen Copenhagen Denmark; ^5^ Department of Dermatology Hospital del Mar Research Institute. Universitat Pompeu Fabra Barcelona Spain; ^6^ Faculty of Health Sciences McMaster University Hamilton Ontario Canada; ^7^ LEADER Research Inc. Hamilton Ontario Canada; ^8^ Dr. D.Y. Patil Medical College & Hospital Mumbai India; ^9^ Department of Dermatology University Hospital Zurich Zurich Switzerland; ^10^ Faculty of Medicine University of Zurich Zurich Switzerland; ^11^ Universidad de Guayaquil Guayaquil Ecuador; ^12^ Hospital Solca Núcleo de Tungurahua Ambato Ecuador; ^13^ EPHORA Research Group; ^14^ Sanatorio Parque Rosario Argentina; ^15^ Hospital San Angel Inn Chapultepec Ciudad de Mexico Mexico; ^16^ Allergy Department Clinica San Felipe Lima Peru; ^17^ PERUCARE Clinica Angloamericana Lima Peru; ^18^ Department of Dermatology Calcutta National Medical College Kolkata West Bengal India; ^19^ First Moscow State Medical University Moscow Center of Allergy and Immunology Clinical Hospital 52 Ministry of Moscow Healthcare Moscow Russia; ^20^ Department of Pharmacology Dr. DY Patil Medical College Navi Mumbai India; ^21^ Department of dermatology Faculdade de Medicina do ABC Santo André Brazil; ^22^ Division of Allergy Department of Pediatrics Clinical Immunology and Rheumatology Urticaria Center of Reference and Excellence (UCARE) Federal University of São Paulo São Paulo Brazil; ^23^ Department of Internal Medicine Immunology Service Hospital Universitário Clementino Fraga Filho Rio de Janeiro Brazil; ^24^ Urticaria Center of Reference and Excellence (UCARE) University of São Paulo São Paulo Brazil; ^25^ Federal University of Paraná Parana Brazil; ^26^ Urticaria Center of Reference and Excellence (UCARE) Instituto de Alergia e Inmunologia del Sur Buenos Aires Argentina; ^27^ Universidad Estatal de Milagro Cdla. Universitaria “Dr. Rómulo Minchala Murillo”—km. 1.5 vía Milagro—Virgen de Fátima Milagro Guayas Ecuador; ^28^ Ecuadorian Development Research Lab Daule Guayas Ecuador; ^29^ Department of Dermatology Faculty of Medicine Pontificia Universidad Católica de Chile Santiago Chile; ^30^ University Dermatological Center (DermaCDU) Las Condes Chile; ^31^ Unit of Dermatology Rancagua Regional Hospital Rancagua Chile; ^32^ Centro de Alergia Tinoco Machala Ecuador; ^33^ The Centre of Positive Psychology and Health Royal College of Surgeons Dublin Ireland; ^34^ Department of Dermatology Koç University School of Medicine Istanbul Turkey; ^35^ Fraunhofer Institute for Translational Medicine and Pharmacology ITMP Immunology and Allergology Berlin Germany; ^36^ Psychodermatology Department Chair of Pulmonology Rheumatology and Clinical Immunology Medical University of Lodz Lodz Poland

**Keywords:** alexithymia, chronic urticaria, demographics, predictors, Toronto alexithymia scale

## Abstract

**Introduction:**

The relationship between chronic urticaria (CU) and alexithymia, a cognitive‐affective impairment characterized by difficulty in identifying and expressing emotions, is complex and underexplored. This study aimed to identify predictors of alexithymia in CU patients by focusing on the impact of coexisting mental illnesses and antihistamine use.

**Methods:**

An online survey was distributed to specialized allergy and dermatology centers from 2021 to 2022. The survey included the TAS‐20, UAS‐7, UCT, CU‐Q2oL, and demographic information. Participants were 18–80 years old, diagnosed with CU, and had no prior diagnosis of alexithymia. The final analysis included a total of 332 respondents from various countries. Regression models were used to investigate the relationship between clinical and demographic factors of patients with CU as key predictors of alexithymia.

**Results:**

Among CU patients, the main predictors of having alexithymia were: presenting mental (OR = 2.406, *p* < 0.05) and cardiovascular comorbidities (OR = 2.085, *p* < 0.05), active urticaria (as opposed to being urticaria‐free), OR = 1.989, *p* < 0.05, severe impact on quality of life (OR = 1.973, *p* < 0.01), and the use of oral first‐generation antihistamines (OR = 2.340, *p* < 0.05). The duration of chronic urticaria diagnosis and other types of treatments (sg‐AH use, omalizumab use, and corticosteroid use) do not appear to be significantly associated with alexithymia.

**Conclusions:**

Alexithymia is closely linked to clinical and demographic variables among patients with CU. These findings suggest that comprehensive management of CU should include psychological assessment and support, especially for patients with alexithymia and those using fg‐AH. Reducing the reliance on fg‐AH and addressing mental health issues may improve outcomes for these patients.

## 1 INTRODUCTION

Stokes was the first to describe the relationship between mental health and inflammatory skin disorders in 1940.^1^ Thirty years later, Nemiah and Sifneos described a subclinical cognitive‐affective impairment affecting the ability to describe individuals' experiences and emotions, which received the name alexithymia.^2^ Emotion regulation is a major issue in people with alexithymia due to a decreased ability to express and recognize one's emotions.^3^

Chronic urticaria (CU) is a persistent inflammatory and debilitating skin condition marked by recurring wheals (hives) and/or angioedema (swelling) for 6 weeks or more,^4^ affecting 1.5% of the global population.^4^, ^5^ CU can severely impair the quality of life by causing stress, sleep disturbances, low self‐esteem, social difficulties, and negative emotions such as anger and despair.^6^ A meta‐analysis revealed that up to one‐third of CU patients also have a mental health condition due to a complex neuro‐immune‐cutaneous‐endocrine network,^7^ including alexithymia.^8^ Alexithymia can amplify somatic symptoms and heightened symptom severity,^8^, ^9^ thereby affecting how chronic diseases are perceived and managed.^10^

Cherrez et al. previously reported a 42% prevalence of alexithymia among CU patients.^10^ However, identifying strong predictors of alexithymia remains underexplored, with existing studies yielding contradictory results. Salminem et al. found associations between alexithymia and male sex, advanced age, low education, and low socioeconomic status among Finns.^11^ Maniaci et al. identified a positive correlation between CU and depressive characteristics but found no interaction between depressive patterns and alexithymia.^12^ Conversely, Barbosa et al. reported no significant statistical correlations between alexithymia and clinical variables.^13^

Recognizing the connection between alexithymia and CU is essential as promptly identifying the primary risk factors and initiating early management could significantly impact the outcomes of controlling and managing these conditions. Therefore, this study aims to examine the characteristics of patients with CU and alexithymia to identify the primary factors that contribute to the development of alexithymia, with a specific emphasis on the influence of coexisting mental illnesses and the use of antihistamines. Finally, the study aims to provide valuable insights into the impact of alexithymia on CU, including its severity and control.

## 2 METHODS

### 2.1 Data collection

This is a cross‐sectional study that included an online survey that was distributed to specialized and referral allergy and dermatological centers that are members of the Urticaria Research Network between 2021 and 2022. Every center administrator who participated disseminated the online survey to their physicians who treat CU. The latter randomly selected CU patients who met the inclusion criteria to fill out the surveys. The inclusion criteria for our participants were as follows: (1) between 18 and 80 years old, (2) without diagnosis of alexithymia, (3) willing to undergo a clinical evaluation and complete a self‐administered questionnaire during the study.

### 2.2 Analytical sample

We initially surveyed 423 patients; 91 patients (22%) that had “possible alexithymia” were not included in the analysis. The final sample of 332 participants included various countries, with Russia having the highest representation at 36%, followed by Brazil at 24%, and Ecuador at 23%, Peru 8%, Mexico 4%. The remaining 5% comprised participants from Denmark, Spain, Argentina, Switzerland, Colombia, Italy, Bolivia, Canada, France, Germany, Pakistan, Sweden, and Tibet.

### 2.3 Questionnaire

Participants were asked to complete a battery of self‐report questionnaires, including the TAS20 (Toronto Alexithymia Scale), UAS‐7 (Urticaria Activity Score‐7), UCT (Urticaria Control Test), CU‐Q2oL (Chronic Urticaria Quality of Life), and demographic information. Medical providers were also asked to supply details on their patients' medical history, including comorbidities, urticaria medication type, years since diagnosis, and other pertinent medical information.

### 2.4 Ethics review

The study was conducted following the ethical principles outlined in the World Medical Association Declaration of Helsinki and received approval from Ecuador's Clínica Kennedy IRB in Ecuador (approval number HCK‐CEISH‐19‐0059). Before their participation, all participants provided informed consent, ensuring that they were fully aware of the nature and purpose of the study. To ensure the anonymity and protection of the participants' information, personal identification was not collected in the study, preserving participant privacy, and ensuring the ethical conduct of the study.

### 2.5 Outcome variable

The twenty‐item Toronto Alexithymia Scale (TAS‐20) assessed alexithymia.^14^, ^15^ Scores ≤51 indicated no alexithymia, while scores ≥61 indicated alexithymia. Scores of 52–60 were classified as “possible alexithymia,” however this was not included in the analysis. Thus, the primary outcome variable was categorized as the presence or absence of alexithymia.

### 2.6 Main predictor variables

The present study considered several control variables, including the CU activity, the impairment of quality‐of‐life domains, use of antihistamines fg‐AH (first‐generation antihistamine), sg‐AH (second‐generation antihistamine), omalizumab, and corticosteroids. We collected these variables through self‐administered questionnaires and medical records.

The presence of mental disease comorbidities was recorded using a dichotomous variable, where the presence was coded as “1” and its absence was coded as “0.” The use of medications was also recorded using a dichotomous variable, where the use of each medication type was coded as “1,” and its nonuse was coded as “0.”

### 2.7 Statistical analysis and modeling

We calculated descriptive statistics for all model variables and employed ordinal regression analysis to model the relationship between alexithymia and chronic urticaria, using TAS‐20 as the main outcome variable reported in a binary manner. We created different models with three standardized and validated Chronic Urticaria PROMs as predictors: UCT, UAS7, and CUQ2OL. The Brant test of proportionality confirmed that all models complied with the test, and by using standardized PROMs, objective measures, and multivariate analyses, we aimed to minimize bias and confounding in our study. Our sensitivity analysis showed consistent coefficients across different levels of CU activity, resulting in a comprehensive and reliable assessment of the relationship between CU and alexithymia. All statistical analyses were performed using Stata 17.0.

## 3 RESULTS

### 3.1 Main descriptive results

Table 1 presents the main descriptive results of the CU patients analyzed. Most participants were female (*n* = 189, 57%) with a mean age of 42.4 years. Chronic spontaneous urticaria (CSU) was the predominant subtype (*n* = 196, 59%). The average disease duration was 59.3 months. Overall, the mean scores for UCT, UAS7, and CUQ2oL were 12.2 (controlled), 14.0 (mild activity), and 51.0 (severe impact on quality of life), respectively. The most common comorbidities were allergy disorders and cardiovascular diseases, accounting for 33% and 20%, respectively. In general, 88% of CU patients received oral sg‐AH.

**TABLE 1:** Descriptive characteristics of chronic urticaria patients by alexithymia status (yes/no) measured by TAS‐20.

	No alexithymia (*n* = 154)		Alexithymia (*n* = 178)		*p* value	Overall (*N* = 332)
	*N*	%	*N*	%		*N*
Sex					0.088^+^	
Male	74	51.7	69	48.3		143
Female	80	42.3	109	57.7		189
Age		43.6		41.2	0.152	
Country					0.010*	
Ecuador	20	26.7	55	73.3		75
Argentina	3	42.9	4	57.1		7
Brazil	40	50.0	40	50.0		80
Colombia	0	0.0	2	100.0		2
Peru	15	60.0	10	40.0		25
Bolivia	0	0.0	1	100.0		1
Mexico	12	60.0	8	40.0		12
Canada	1	100.0	0	0.00		1
France	0	0.0	1	100.0		1
Germany	0	0.0	1	100.0		1
Russia	63	52.9	56	47.1		119
Type of CU					0.773	
CSU	94	48.0	102	52.0		196
CIndU	18	42.9	24	57.1		42
Both spontaneous and inducible	42	44.7	52	55.3		94
CU diagnosis time (months)		62.5		56.1	0.484	
UCT category					0.014*	
Uncontrolled	44	37.3	74	62.7		118
Controlled	110	51.4	104	48.6		214
Mean score		12.9		11.6	0.001**	12.2
UAS7 interpretation					0.006**	
Urticaria‐free	40	61.5	25	38.5		65
With activity of CU	114	42.7	153	57.3		267
Mean score		12.2		15.6	0.017*	14.0
CUQ2OL domain (0–100)						
Functioning domain		22.1		32.6	0.000***	
Sleep domain		30.2		41.6	0.000***	
Itching embarrassment domain		30.5		41.6	0.000***	
Mental status domain		26.9		44.7	0.000***	
Swelling/Eating domain		16.9		24.6	0.003**	
Limits‐looks domain		18.6		29.8	0.000***	
Mean score		45.4		55.8	0.000***	51.0
Comorbidities						
Cardiovascular comorbidity	24	35.8	43	64.2	0.052^+^	67
Autoimmune comorbidity	19	46.3	22	53.7	0.995	41
Cancer comorbidity	3	42.9	4	57.1	0.850	7
Allergy comorbidity	45	40.5	66	59.5	0.130	111
Mental disease comorbidity	10	25.6	29	74.4	0.006**	39
Treatment						
Oral fg‐AH use	12	27.9	31	72.1	0.009**	43
Oral sg‐AHuse	131	44.9	161	55.1	0.133	292
Omalizumab use	40	48.2	43	51.8	0.703	83
Corticosteroids use	14	41.2	20	58.8	0.520	34

*Note*: Differences in statistical significance between means or frequencies were assessed using either ANOVA or chi‐squared tests, depending on whether the variable was continuous or categorical.

Abbreviations: CIndU, chronic inducible urticaria; CSU, chronic spontaneous urticaria; CU, chronic urticaria; CUQ2OL, chronic urticaria quality of life questionnaire; fg‐AH, first‐generation antihistamines; sg‐AH, second generation antihistamines; TAS‐20, Toronto alexithymia scale‐20; UAS7, urticaria activity score in 7 days; UCT, urticaria control test.

Significance levels are indicated by the following symbols: ***p < 0.001, **p < 0.01, *p < 0.05, + *p* < 0.1.

When comparing the alexithymia (*n* = 178) and non‐alexithymia (*n* = 154) groups, the demographic distribution was significantly different between them. Patients without alexithymia have a higher mean UCT score (12.9) compared with those with alexithymia (11.6). This difference is statistically significant with a *p*‐value of 0.001. A higher percentage of patients with alexithymia (62.7%) had uncontrolled urticaria compared to those without alexithymia (37.3%). Individuals with alexithymia have a significantly higher mean UAS7 score (15.6) compared with those without alexithymia (12.2), with a *p*‐value of 0.017. A greater proportion of patients without alexithymia (61.5%) were urticaria‐free compared with those with alexithymia (38.5%). In terms of quality of life, there is a higher mean score (55.8) on the CU‐Q2OL, indicating worse quality of life in the alexithymia group compared to those without alexithymia (45.4). A higher percentage of patients with alexithymia (41%) reported a severe impact on their QoL compared to those without alexithymia (24%). These differences were statistically significant with a *p*‐value <0.001.

The alexithymia group had a higher prevalence of comorbidities, though only mental disease comorbidity was statistically significant, 74% in the alexithymia group versus 26% in the non‐alexithymia group (*p* 0.006). Treatment types showed significant differences among the alexithymia group; 72% used oral fg‐AH versus 28% in the non‐alexithymia group. The most common comorbidities were allergy disorders and cardiovascular diseases, accounting for 33% and 20%, respectively.

When focusing on the influence of coexisting mental illnesses and the use of antihistamines, we found that 26% of participants with mental disease comorbidities used fg‐AH medications, compared with 11% without such comorbidities (Table S1).

### 3.2 Regression results

The bivariate logistic regression analysis presented in Table 2 evaluates various risk factors associated with the presence of alexithymia in patients with CU, highlighting several significant predictors of alexithymia including mental (OR = 2.406, 95% CI 1.058–5.461, *p* < 0.05) and cardiovascular comorbidities (OR = 2.085, 95% CI 1.113–3.907, *p* < 0.05), active urticaria (as opposed to being urticaria‐free), OR = 1.989, 95% CI 1.087–3.642, *p* < 0.05, severe impact on quality of life (OR = 1.973, 95% CI 1.183–3.289, *p* < 0.01), and the use of oral first‐generation antihistamines (OR = 2.340, 95% CI 1.090–5.020, *p* < 0.05). The duration of chronic urticaria diagnosis and other types of treatments (sg‐AH use, omalizumab use, and corticosteroid use) do not appear to be significantly associated with alexithymia. To identify the association between mental disease comorbidities and the use of first‐generation antihistamines, the bivariate logistic regression indicated a positive association between mental disease comorbidities and fg‐AH use (OR = 2.717, 95% CI 0.195–1.804, *p* = 0.015) (Table 3).

**TABLE 2:** Binary logistic regression analysis of factors associated with alexithymia (yes/no) in chronic urticaria patients.

Predictor variables	Outcome = alexithymia
Comorbidities	
Mental disease	2.406* (1.058–5.461)
Cardiovascular disease	2.085* (1.113–3.907)
Chronic urticaria diagnosis time (months)	0.998 (0.995–1.001)
UAS7	
Urticaria‐free	(Base outcome)
With activity of CU	1.989* (1.087–3.642)
CUQ2OL	
Moderate impact of QoL	(Base outcome)
Severe impact of QoL	1.973** (1.183–3.289)
Type of treatment	
Oral first‐generation antihistamines use	2.340* (1.090–5.020)
Oral second‐generation antihistamines use	1.496 (0.732–3.056)
Omalizumab	1.007 (0.588–1.564)
Corticosteroids	0.586 (0.257–1.334)
/cut1	3.196** (1.361–7.510)
Observations	332

*Note*: CI in parentheses.

***p < 0.001, **p < 0.01, *p < 0.05, + *p* < 0.1.

**TABLE 3:** Binary logistic regression analysis evaluated the association between mental disease comorbidities and the use of first‐generation antihistamines.

Predictor variables	Outcome = Mental disease comorbidity
Oral first‐generation antihistamines use	
No	(Base outcome)
Standard dose	2.717*
	(1.215–6.076)
/cut1	8.966***
	(6.107–13.151)
Observations	332

*Note*: CI in parentheses. The bivariate logistic regression analysis of the relationship between mental disease comorbidities and the use of first‐generation antihistamines indicated a positive relationship, meaning that the use of fg‐AH roughly tripled the odds of mental health comorbidities compared to those who did not use it (OR = 2.717, 95% CI 1.215–6.076, *p* = 0.015).

***p < 0.001, **p < 0.01, *p < 0.05, + *p* < 0.1.

### 3.3 Predicted probabilities of the two categories of the TAS‐20

**FIGURE 1:**
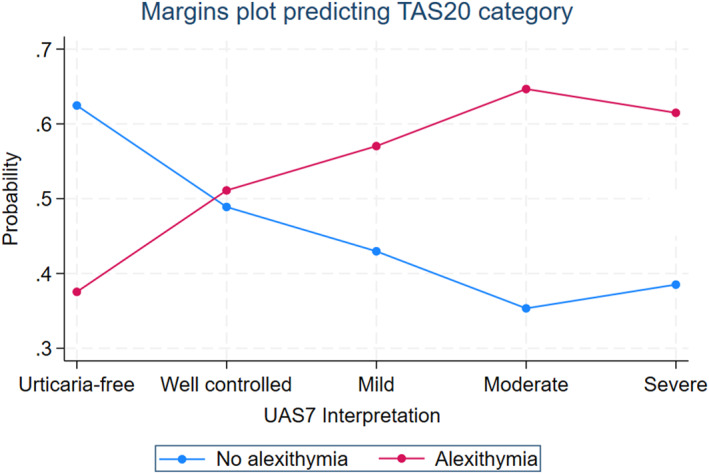
Margins from ordinal logistic regression predicting the TAS20 category as a function of UAS7 interpretation categories.

Figure 1 depicts the predicted probabilities of TAS‐20 based on UAS7 interpretation, holding other variables constant, and elucidates UAS7 prediction cutoff points. The red line shows a 39% alexithymia probability for urticaria‐free patients (UAS7 = 0), while those with well‐controlled to severe CU activity had a 51%–65% probability.

## 4 DISCUSSION

In general, our study found that there were significant predictors that increased the likelihood of alexithymia among CU patients, which included active urticaria, severe QoL impact, mental or cardiovascular comorbidities and H_1_ antihistamine use.

Our findings contradict Barbosa's descriptions of the relationship between activity and control of CU and alexithymia. They did not observe any interaction between clinical factors.^13^ However, in terms of quality of life, Mattila indicated that alexithymia had a negative correlation with the overall health‐related quality of life in the general population.^16^ In specific dermatologic disorders (i.e. hidradenitis suppurativa,^17^ acne^18^), there have been reports of significant correlations with poor quality of life.

When analyzing the relationship between mental comorbidities and risk of alexithymia, it is important to highlight that the skin is a target and source of stress mediators in a bidirectional manner.^19^ Tomaszewska et al.^20^ identified CU under a stress‐modulated condition, with increased disease severity linked to higher psychological distress and vice versa.^21^ A systematic review estimated the prevalence of psychiatric comorbidities among CU patients, regardless of control groups, at 32%.^22^ Anxiety and depression were found in over 40% of urticaria patients in case‐control and cross‐sectional studies.^23^, ^24^ Additionally, alexithymia was present in more than 40% of patients,^10^ indicating a significant mental health burden. Interventions are necessary to alleviate chronic stress, which at the cellular level, perpetuates inflammatory mediators, interleukins, and neurogenic inflammation, possibly exacerbating CU activity and control.^20^, ^25^

The mechanisms are not entirely clear, but alexithymia, anxiety, and depression are hypothesized as triggers for CU,^6^, ^22^, ^26^ possibly due to the interaction between the immune and central nervous systems (CNS).^27^ The immune response in people with alexithymia is marked by increased glucocorticoid production, reduced cell‐mediated immunity, and a Th1/Th2 imbalance favoring Th2, which are also involved in dermatologic disorders.^28^, ^29^, ^30^ These individuals often exhibit hypervigilance and hyperarousal in response to threats,^31^ supporting the “stress‐alexithymia hypothesis” that suggests alexithymia's cognitive, behavioral, and physiological aspects may contribute to stress‐related illnesses like CU.^32^ However, it is worth considering whether the reverse could be true, or if these phenomena occur as part of the disease process. All of these can be alleviated by appropriate management of CU; this is a question that will be answered with high‐quality studies in the future. Additionally, the relationship between alexithymia and CU has been examined regarding psychological comorbidities and posttraumatic stress, highlighting the emotional factors in the manifestation and management of this skin condition.^33^

In our sample, 11.7% had a mental disease comorbidity, increasing the risk of alexithymia 2.4 times. It is recommended to regularly evaluate the effectiveness of the CU strategy in clinical practice using standardized and validated patient‐reported outcomes (PROs). This includes assessing the impact on the quality of life of patients with CU, particularly in the mental health domain. The Chronic Urticaria Quality of Life (CU‐Q2oL) questionnaire is useful for this purpose as it includes items related to mental well‐being.^6^, ^34^, ^35^ We suggest that more than CU‐Q2oL, specialists could use mental health questionnaires such as: Hospital Anxiety and Depression Scale,^36^ Beck Depression Inventory,^37^ TAS‐20,^14^ among other questionnaires that might help us to suspect mental problems.

The evidence provided here suggests that alexithymia may be associated with CU, which raises the question of whether recognizing and addressing alexithymia may improve CU. Alexithymia may be reversible, and reversion has been associated with improved measures of disease severity, psychological comorbidities, work productivity, and QoL in dermatologic patients.^30^ A multimodal therapeutic strategy that includes prompt recognition and management of suspected psychological issues is needed. Additionally, psychotherapeutic and pharmacological treatment for anxiety disorders and alexithymia in patients with past CU may prevent the recurrence of urticaria symptoms.^7^, ^38^, ^39^ For example, the Irish Attention‐based training (ABT) program^32^ aims to improve CSU patient outcomes through systematic attention‐focused practice.^40^, ^41^

### 4.1 First‐generation antihistamines doubled the risk of alexithymia

H_1_ antihistamines are not recommended in CU due to serious side effects and drug interactions.^42^, ^43^ International Guidelines suggest sg‐AH as the first‐line treatment.^44^ Nevertheless, our findings revealed that specialists still prescribe fg‐AHs, doubling the risk of alexithymia. We investigated a potential link between fg‐AH use and mental health comorbidities, noting that some healthcare providers might prescribe fg‐AH to aid sleep in patients with anxiety/depression and CU. Our results showed that 2 out of every 10 participants with mental disease comorbidities used these medications, and fg‐AH increased the likelihood of presenting mental disease comorbidities. In concordance with our results, Ozdemir et al. found that patients who received cetirizine and hydroxyzine treatments reported higher scores on the depression, anxiety, and fatigue sub‐scales than those who received desloratadine, levocetirizine, and rupatadine.^45^

Although there is no clear pathway linking alexithymia to fg‐AH drugs, the functional properties of the H1 receptor include regulation of cell proliferation and differentiation, hematopoiesis, embryonic development, regeneration, and wound healing, and they play an important role in neurotransmission in the CNS, behavioral state and reinforcement (novelty, alertness), learning and memory, feeding rhythms, energy metabolism, and endocrine control. The underlying mechanisms of alexithymia are involved in the onset of sleep disorders, mood changes, memory problems, eating disorders, addiction, pain, and neuroinflammation. These conditions do not necessarily correlate with sedation, drowsiness, fatigue, or sleepiness.^42^, ^46^

This novel finding implies a potential correlation between this drug class and alexithymia, warranting further investigation. Our results recommend routine mental health evaluations for CU patients. A multidisciplinary approach is essential to help patients manage symptoms and to identify and treat any mental conditions, thereby enhancing their health and QoL.

## 5 LIMITATIONS

Our study has several limitations. First, the sampling strategy did not encompass all individuals with CU at each study site, limiting the generalizability of the results. This expert sampling, which gathered data from global medical providers specializing in CU, may introduce selection bias. Second, as a cross‐sectional study, it cannot establish causality between alexithymia and CU. Longitudinal studies are needed to explore the temporal relationship between these variables. Future research should include randomized clinical trials to investigate therapeutic approaches targeting alexithymia to improve disease outcomes and patients' quality of life, as well as the association between FG‐ATH1 and alexithymia.

## 6 CONCLUSION

The high prevalence of alexithymia in CU is linked to high activity and poor control of the disease. The causal direction of this relationship is unclear and requires further investigation: does CU increase alexithymia traits or does alexithymia worsen CU symptoms? Addressing alexithymia in CU patients could aid in developing personalized treatment strategies that consider the psychological aspects of the condition. Comprehensive management strategies should address both dermatological and psychological well‐being, advocating for a multidisciplinary approach to urticaria treatment. Such strategies may be formulated by recognizing and treating alexithymia along with CU symptoms.

## AUTHOR CONTRIBUTIONS

**Ivan Cherrez Ojeda**: Conceptualization, investigation, funding acquisition, writing—original draft, methodology, writing—review and editing, supervision, resources. **Simon Francis Thomsen**: Writing—original draft, methodology, validation; investigation, writing—review and editing. **Ana Gimenez‐Arnau M**: Methodology, validation, investigation, writing—review and editing. **Jennifer Astrup Sorensen**: Writing—review and editing, investigation, resources. **Hermenio Lima**: Writing—original draft, writing—review and editing, resources. **Kiran Godse**: Investigation, validation, writing—review and editing. **Carole Guillet**: Investigation, validation, writing—review and editing. **Luis Escalante**: Investigation, resources, writing—review and editing. **Astrid Maldonado**: Writing—original draft, investigation; methodology. **Gonzalo Federico Chorzepa**: Investigation, writing—review and editing, resources. **Blanca Morfin‐Maciel**: Writing—review and editing, investigation, resources. **Jose Ignacio Larco Sousa**: Writing—original draft, writing—review and editing, resources. **Erika de Arruda‐Chaves**: Writing—original draft, writing—review and editing; supervision, investigation. **Abhishek De**: Writing—review and editing, writing—original draft, investigation, resources. **Daria Fomina**: Writing—review and editing, writing—original draft, investigation. **Anant Patil**: Writing—review and editing, writing—original draft, investigation. **Roberta Jardim Criado**: Writing—review and editing, writing—original draft, investigation. **Luis Felipe Ensina**: Writing—review and editing, writing—original draft, investigation. **Solange Valle O R**: Writing—original draft, investigation, writing—review and editing. **Rosana Camara Agondi**: Writing—original draft, writing—review and editing; resources. **Herberto Chong Neto**: Investigation, writing—review and editing, resources, methodology. **Nelson Rosario**: Investigation, writing—review and editing. **German Dario Ramon**: Investigation, writing—original draft, writing—review and editing, resources. **Marco Faytong‐Haro**: Data curation, software, formal analysis, project administration, validation, methodology, supervision. **Isabel Ogueta**: Funding acquisition, writing—original draft, supervision. **Ivan Tinoco Moran**: Investigation, writing—original draft, writing—review and editing, resources. **Jennifer Donnelly**: Writing—review and editing, writing—original draft, investigation. **Emek Kocatuerk**: Resources, supervision, formal analysis, writing—review and editing, writing—original draft, investigation. **Anna Zalewska‐Janowska**: Writing—review and editing, writing—original draft, methodology, formal analysis, investigation. **Karla Robles‐Velasco**: Conceptualization, investigation, writing—original draft, writing—review and editing, supervision, resources, project administration, methodology, validation.

## CONFLICT OF INTEREST STATEMENT

EK declares being a speaker and advisor for Novartis, Menarini, LaRoche Posey, Sanofi, Bayer, Abdi İbrahim, Pfizer. SFT declares being speaker or advisor for AbbVie, Almirall, Boehringer, Eli Lilly, Galderma, Incyte, Janssen Pharmaceuticals, LEO Pharma, Novartis, Pfizer, Sanofi, UCB Pharma, and Union Therapeutics, and received research support from AbbVie, Janssen Pharmaceuticals, LEO Pharma, Novartis, Sanofi, and UCB Pharma outside the submitted work. The rest of the coauthors report no conflicts of interest.

## Supporting information

Table S1

## Data Availability

The datasets used and/or analyzed during the current study are available from the corresponding author on reasonable request.
