# Supplementary material for: Exploring the impact of chronic urticaria profile as a key predictor of alexithymia: A cross‐sectional study
Source: Clin Transl Allergy. 2025 Jul 4;15(7):e70075. doi: 10.1002/clt2.70075 (PMC12227325; doi:10.1002/clt2.70075)
Supplement: Supplementary file 1 — Table S1 [file CLT2-15-e70075-s001.docx]

**Supplemental Table S1**. Comparison of Use of First-Generation Antihistamines Between Chronic Urticaria Patients with and without mental disease comorbidities

|  | Mental Disease Comorbidity  No  (n= 293) | | Mental Disease Comorbidity  Yes  (n= 39) | | p value |
| --- | --- | --- | --- | --- | --- |
|  | N | % | N | % |  |
| Fg-AH |  |  |  |  | 0,012 |
| No | 260 | 88.74% | 29 | 74.36% |  |
| Standard dose | 33 | 11.26% | 10 | 25.64% |  |

Fg-AH: First-generation Antihistamine-1-receptor.

Note: Differences in statistical significance between frequencies were assessed using chi-squared test.
